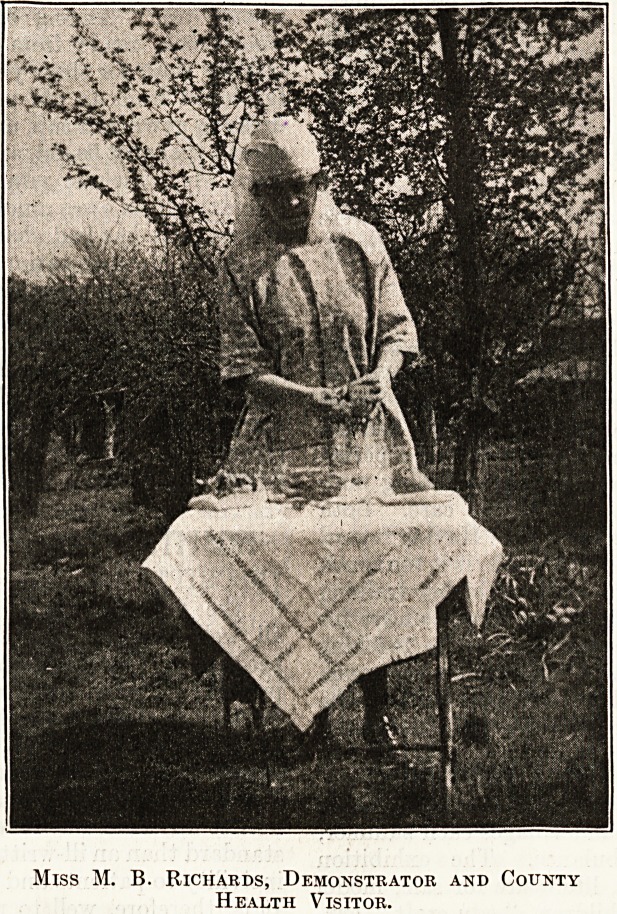# The Art of Living

**Published:** 1923-11

**Authors:** H. S. Cooper Hodgson

**Affiliations:** (Superintendent of Health Visitors to Durham County Council)


### THE ART OF LIVING.

### THE WORK OF THE HEALTH VISITOR.

By Miss H. S. COOPER HODGSON (Superintendent of Health Visitors to Durham County Council).

THE health visitor's business is to teach the art of living. The population of these isles has more than quadrupled itself within the last hundred years. How changed is England from the days when each small community had its bootmaker, weaver, and carpenter; when a man might well have known by sight the beast from whose hide his boots were fashioned, the sheep whose wool clothed him, and the very trees from which his chairs and tables were made. Small possibility in such circumstances of bad materials and less of skimped work.

### The Lost Power of Discrimination.

With our great population we are obliged to have factory-made goods. But we need not have this present flood of inferior stuff—all the "ines" and "ettes," "best strawberry jam improved with fruit juice," boots made on the "hand-sewn principle," "solid" mahogany chests of drawers of which a reputable citizen is not ashamed to tell a county court judge that "in the trade" "solid mahogany" implies that the larger part of the article is of an inferior wood. Nor need we have all the potted pastes — "pheasant," "turkey and tongue," "chicken" and bloater—the humble bloater being the most highly priced of the lot! Apart from the financial stringency which, we hope, is passing away, a main source of trouble is that dwellers in both large and small houses have lost their powers of discrimination, buying rubbish because they don't know any better.

Miss M. B. Richards, Demonstrator and County Health Visitor.

### Fighting Social Evils.

Leaving the big house folk to fight the matter out for themselves, for they are at school until twenty and can learn if they want to, I am mainly concerned with homes where every misspent penny means somebody going short of an essential. The children learn at school, but leave at fourteen; even if their small minds could absorb all that goes to the art of living, let us have pity and remember that we ourselves were at that tender age. Would our parents have remodelled their way of life in accordance with suggestions brought home by us? It is more than likely that our well-meant advice might have met a quite ungracious reception. Since then, however, the health visitor has been created, and she had no sooner come into being than she found her hands full with innumerable social evils to combat—unsuitable food and drinks, not only for the baby, but for all the members of the family; unsuitable clothing for all of them; wrong health habits; ill-ventilated as well as bad houses; carelessly used as well as unsuitable closets, ashpits, and backyards.

### Women of all Work.

We, in the public health department, are face to face with the most pressing social problems — we cannot escape them; every evil we combat has its root in social conditions. Almoning, milk assistance, free or assisted midwifery, hospital and convalescent home treatment, and home helps have recently been added to our duties, all part of the transference of the necessitous mother and young child from the care of the Poor Law to that of the Health Department, foreshadowed by the report of the Poor Law Commission of 1906—part, too, of the much-discussed, and inevitably coming, family endowment scheme. Durham County health visitors have, in addition to infant visiting, the visiting of tubercular cases, following up of defective school children, care of mental defectives (an intricate problem, the menace of which to the community is as yet barely realised), and welfare work for the blind; the management of the travelling welfare exhibition which visits about twenty places each summer, and of the "Welfare Journal," which has a circulation of 2,000. We staff forty clinics—infant, dental, tuberculosis, and venereal disease—each week. There is not a dull moment.

### Varied Activities.

Desiring to have a celebrated London lecturer heard in the county, the health visitors last May obtained the co-operation of the local Citizens' League, sold half-crown tickets, collected an audience of several hundred people from all parts of the county, cleared the expenses (£20), and handed over a balance of £2 10s. to a worthy object. In 1922 the staff undertook Group Studies, largely as the result of observations of certain lecturers at the first Winter School for Health Visitors, particularly Dr. Sloan Chesser, Dr. H. C. Cameron, and Miss E. V. Eckhard, on the great need for research work in social circumstances by health visitors. There are two reading circles, which held debates; the Exhibition Committee organised demonstrations in housewifery, cooking, and easy dressmaking; three of the staff, with special training in the care of mental defectives (obtained under our half-salary study leave scheme, through the kindness of Miss Evelyn Fox, of the Central Association for Mental Welfare), made special studies of 300 mental defectives under supervision (half of the total number). They discovered some interesting facts. For instance, six individuals were married and had among them twenty-three children, of whom several were mental defectives.

### Training and Pay.

The training of the health visitor is still the subject of discussion. Since she is a social worker, the social side of her training, including economics, social administration, domestic science, psychology, and the care of mental defectives, must cover as long a period as that devoted to hospital training, including midwifery and modern methods of infant care. Both salaries and training need stabilising. Having regard to the national importance of her work, is it too much to ask that more liberal grants-in-aid of her training be afforded the pupil health visitor, such as have for years been enjoyed by the members of the teaching profession? This is the more important since much of the teacher's work will be nullified without the health visitor's intelligent reinforcement in the home.

### The Travelling Welfare Exhibition.

The County Durham Travelling Welfare Exhibition visits from 20 to 30 towns and villages each summer, travelling by motor ambulance. The exhibition includes about 150 posters, literature for sale, model clothing for infants and children, "edu-craft" garments, facts about food for people of all ages, economy ideas, household hints and training material for mental defectives. There are eight large wooden screens, with properly fitting draperies of soft green and brown, which make an effective background for the posters and exhibits. The posters deserve a word of explanation. Some of them were obtained from the Baby Week Council and other similar sources, including "Advice about Teeth," "Clean Milk Propaganda," and the Ling Posture Posters. The remainder are specially designed; many of them relate to vital statistics of the county. A shaded map taken from a Local Government Board Report, dealing with child mortality at ages 0–5, has created a tremendous amount of interest, and has brought many workers to the Welfare field. Other posters are hand-painted portraits of attractive babies with wise sayings attached to them. There is a large diagram showing what happens when foreign bodies are pushed down drains; a list of "Health Chores," now so much a feature of health propaganda work in American schools; and two diagrams from the Milk Commission Report, showing respectively the human and the cow population of England. There are model cots, a playing pen, and a "Baby Jumper," made by the blinded warriors at St. Dunstan's.

### Some Economical Notions.

In the Economy Section are some useful articles made by an amateur from discarded tins—pastry cutters, flour dredgers, and scoops, pans, candlesticks, lading tins, teapots, and, most popular of all, a little oven for fitting over a gas ring, made from two biscuit tins. Among other exhibits are a baby's wool frock, made from the best pieces of a scarf already worn for three years; an attractive handbag fashioned from an old panama hat; and a summer dressing gown and cap, made from a dyed cotton bedspread that cost 5s. 6d. and served as a quilt for years. Food ideas include vitamins, calories, and balance. The exhibition owes much to my assistant, Miss E. F. Brown, who has had charge of it from the beginning. Practical demonstrations by the County Health Visitors form a popular feature. The demonstrations include, as well as the ever-popular "How to bath the baby," how to make a one-piece dress any woman would be pleased to wear, how to make the new salads, how to make five useful articles from three sheets of brown paper, how to cook cabbage, cheese, and other oddments, and the artistic and hygienic furnishing of the miniature residence.

## Figures and Tables

**Figure f1:**